# Home and community based care program assessment for people living with HIV/AIDS in Arba Minch, Southern Ethiopia

**DOI:** 10.1186/1472-684X-11-8

**Published:** 2012-06-15

**Authors:** Taddese Alemu Zerfu, Yaliso Yaya, Selamawit Dagne, Kebede Deribe, Horacio Ruiseñor-Escudero, Sibhatu Biadgilign

**Affiliations:** 1 Department of Public Health, College of Public Health and Medical Science, Dilla University, Dilla, Ethiopia; 2 Department of Nursing, Arba Minch College of Health Sciences, Arba Minch, Ethiopia; 3 Ethiopian Kale Hiwot Church (EKHC), Arba Minch Medanacts HIV/AIDS Prevention and Control Project Coordination Office, Arba Minch, Ethiopia; 4 Fayyaa Integrated Development Association-NCMI, PEPFAR-New Partners Initiative, P.O. Box 5035, Jimma, Ethiopia; 5 Department of International Health, Johns Hopkins Bloomberg School of Public Health, Baltimore, MD, USA; 6 Department of Epidemiology and Biostatistics, College of Public Health and Medical Science, Jimma University, Addis Ababa, Ethiopia

**Keywords:** Community, Home-Based, Palliative Care, PLWHA, Ethiopia

## Abstract

**Background:**

People Living with HIV/AIDS (PLWHA) require significant care and support; however, most care needs are still unmet. To our knowledge, no studies have described the activities and challenges of care services in Ethiopia. Our objective was to assess the status, shortcomings and prospects of care and support services provided to PLWHA in the town of Arba Minch, Ethiopia, and surrounding areas.

**Methods:**

A cross-sectional quantitative study combined with qualitative methods was conducted in Southern Ethiopia among 226 randomly selected PLWHAs and 10 service providers who were purposively selected. Data was collected using a pre-tested structured interview questionnaire and in-depth interview guideline. Quantitative data was analyzed using SPSS windows based statistical software while qualitative data was analyzed manually using thematic framework analysis.

**Results:**

A total of 226 PLWHAs were interviewed. Socio-economic support (material and income generating activities) was being received by 108 (47.8%) of the respondents, counseling services (e.g. psychological support) were being received 128(56.6%), 144 (63.7%) alleviation of stigma and discrimination as human right and legal support for study participants. Inadequate external financial support, lack of proper referral systems between different care providers were among the reasons identified for the low quality and redundancy of care and support activities. Nonetheless, many opportunities and prospects, including easily accessible care receivers (PLWHA), good political and societal will were also implicated.

**Conclusion:**

Care and support services provided to PLWHAs in the study area are by far lower in terms of coverage and quantity. Strategies for improvement could be facilitated given the observed political will, social support and access to care givers.

## Background

In 2009, an estimated 33.3 million people [31.4-35.3 million] were living with HIV and 2.6 million [2.3 million–2.8 million] people who became newly infected with HIV worldwide. In 33 countries, the HIV incidence has fallen by more than 25% between 2001 and 2009; 22 of these countries are in sub-Saharan Africa [1], the region where the majority of new HIV infections continue to occur. It was estimated that 1.8 million [1.6 million–2.0 million] people became infected in 2009 [1]. The global burden of HIV is heaviest in lower-income countries, where the majority of adults with HIV live [2]. Given the magnitude of the regional epidemic, combined with the lack of adequate health infrastructure and human resources, it is necessary to develop community-based palliative strategies and end-of-life care models that adapt to local needs [3,4]. PLWHAs face stigma, discrimination and other violations of their human rights. Protecting human rights and providing legal services for PLWHA and their families are critical components of HIV/AIDS prevention and care services [2,5]. Furthermore, the HIV/AIDS pandemic has caused extreme hardship in already impoverished populations. Globally, less than one in every three households that have a PLWHA are able to pay for even the most basic health care [6].

Research in several countries across the globe suggests that psychological support, healthcare support, spiritual support, alimentary supplementation and financial support are the core needs of PLWHA [7-9]. There is now a general recognition that comprehensive care across the continuum should be provided to PLWHA through all the stages of infection, with a crucial role for community-home based care activities [10]. The care and support needs of PLWHA and their families can be categorized in four interrelated domains: medical needs, psychological needs, socioeconomic needs, and human rights and legal needs [11]. Major challenges remain in scaling-up Antiretroviral Therapy (ART), income generation and meeting nutritional needs of the rapidly increasing number of affected families and orphans [12-15].

In Ethiopia, hospital bed occupancy rate due to AIDS has reached over 50% in urban hospitals, creating a severe burden to health services in the country. Given the direct and the indirect costs that would be linked to this increment, home care could potentially offer a feasible option for patient care, mobilizing a currently dormant resource. This strategy could also have a potential impact in decreasing stigma and discrimination within the families and communities [16].

Given the dynamic nature of the problem, coupled with limited previous research, we have as a result an incomplete description of the context. There is a gap in the quality as well as in the support activities provided to PLWHA [17]. To our knowledge, there are no studies that have described the activities and challenges of care services in Ethiopia for PLWHA. The objective of this study was to assess the service pattern, challenges and prospects of care and support activities for PLWHA and determine their experiences of services received in the resource-limited area of the Southern Regional State of Ethiopia.

## Methods

### Study settings and participants

We conducted a community based, cross-sectional quantitative study using a standardized questionnaire supplemented by qualitative methods using in-depth interviews with care providers from governmental organizations (GO) and non-governmental organizations (NGO). The study was conducted from November 2008 to January 2009 at Arba Minch town and nearby areas in Southern Ethiopia. Arba Minch is the capital of Gamo Gofa Zone, located approximately 500 km to the South of Addis Ababa and 275 Kms away from the Regional capital, Awassa. In the Arba Minch and nearby areas, there are five associations for PLWHA with an estimated of 937 members; of this 302 (32.2%) are males and 635 (67.8%) are females [18]. The study used both quantitative and qualitative methodology. Our sample was taken from PLWHA currently living in Arba Minch town and nearby rural Kebeles (the lowest administrative unit) who had disclosed their HIV sero-status, who could read and write and that had the cognitive capacity to understand what is needed from palliative and home-based care services and to communicate these needs. We excluded PLWHA under 15 years of age. Participants in qualitative interviews were selected purposively and interviewed from six governmental organizations (GO) and four non-governmental organizations (NGO) involved in providing care and support activities.

### Sample and procedure

For the quantitative part of the study, individual PLWHAs in the town and nearby Kebeles were sampled using Probability Proportional -to- Size (PPS) with multistage stratified cluster. Sample size was calculated using EPI-info version 6 windows based statistical software considering the following parameters : a 50% prevalence of palliative care was allocated to get the maximum sample size (range 44%-50%.), precision of 5%, with a 95% confidence interval; 10% was added to compensate for possible non-response. A total sample size of 228 PLWHA was estimated for survey.

For the qualitative component, interviews were held with heads of different GOs and NGOs working on care and support activities. Initially, we identified all PLWHAs by their association (organization in which they are receiving the service) in the study area and stratified them in to rural and urban town area. In Arba Minch there were three associations for PLWHA: Tesfa Goh, Addis Tesfa (Women’s), Bete- Saida and Addis Hiwot Legna from rural PLWHA Association were taken. Study participants were proportionally allocated by association. The sampling procedure involved a stratified sampling technique. After stratification, clustering and proportional allocation was conducted; random selection of participants using lottery method was carried out.

### Measurements

The dependent variables were getting care and support indicated by socio-economic support, medical and nursing care, human rights support and psychosocial care and support. The independent variables were socio-demographic, type of care, duration of care, barriers to care and support. A structured questionnaire was originally developed in English, translated to Amharic and back translated to English to ensure validity. To make findings comparable, care and support measurements were adopted from Family Health International (FHI) core protocol on care and support for PLWHA [19]. The questionnaire was piloted and the final version of the questionnaire was used for data collection. Variables on each item were stigma and discrimination: - material support, medical and nursing care, psychosocial support and human rights and legal support were included on the questionnaire to achieve the objective of the study. Both interviewers and supervisors had received two day training on data collection and care and support issues and research with human subjects. The place of interview was selected based on participants’ convenience. The principal investigator made the interviews by using interview guides for the in-depth interviews. The interviews were tape recorded after obtaining verbal informed consent. The recorded interviews were transcribed first to the original language of the interview and then fully translated into English. Responses and comments were grouped according to the topics and finally writing up and description was performed. Ethical clearance was obtained from the Research and Publication office of the Arba Minch College of Health Sciences Ethical Clearance Committee. Permissions were also obtained from each participating association of PLWHA. Verbal informed consent was also obtained from each individual respondent during data collection.

### Data analysis and processing

Quantitative data was analyzed using SPSS version 16 windows based statistical software while qualitative data was analyzed manually using thematic framework analysis after gathering different data that appeared commonly and grouped under theme. Exploratory data analysis was conducted for socio- demographic and other study variables. The quantitative findings were supplemented to the qualitative findings using triangulation [20]. For data quality assurance, appropriate recruitment and training of interviewers was carried out and monitored frequently in the field and during data entry as well as through close supervision of interviewer. All questionnaires were examined for completeness and consistency during interview.

## Results

### Socio-demographic characteristics of participants

We interviewed a total of 226 (100%) PLWHA, out of which 141 (62.4%) were men. The mean age of the participants was 33.5 ± 6.3 years, with range from 23 to 50 years. A total of 128 (56.6%) respondents were married. Most of the people that were included in our sample, 108 (47.8%), were daily laborers. No formal education was reported by 105 (46.3%) respondents. Regarding religion, 157 (96.5%) reported being Christians (Table 1).

**Table 1:** Socio-demographic characteristics of the study population, Arba Minch town and surrounding areas, 2009 (n = 226)

**Characteristics**		**Frequency**	**Percent (%)**
Sex	Male	85	37.6
	Female	141	62.4
Age	20 – 25	32	14.2
	26 – 30	66	29.2
	31 – 35	58	25.4
	36 – 40	46	20.4
	41 – 45	14	62
	45+	10	44
Marital status	Married	128	56.6
	Single	34	15
	Widowed	38	16.8
	Divorced	22	9.7
	Separated	4	1.8
Occupation	Daily laborer	108	47.8
	House wife	62	27.4
	Merchant	19	8.4
	Government employee	8	3.5
	Others	27	11.9
Education	No formal education	105	46.3
	Primary	75	33.0
	Secondary and above	47	20.7
Religion	Christian	218	96.5
	Muslim	8	3.5

### Types of care and support activities provided to PLWHA

From the comprehensive care and support activities that needs to be provided to PLWHAs, the GOs were mainly involved in the provision of psychosocial, medical and nursing care; while the NGOs were mainly involved in the provision of socio-economic and psychosocial support.

### Socio-economic support

**Figure 1:**
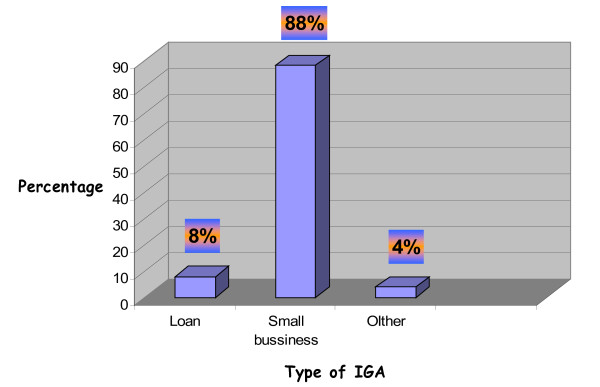
Type of income generating activity in Arba Minch town and Surrounding areas, SNNPR, 2009.

We found that among the various supports that was given to PLWHA, only 58 (25.7%) of the responding PLWHA had obtained material support in the year previous to the start of the study. From those who had obtained the support, a sizable proportion, 38 (65.5%), obtained agricultural materials and seeds, 4 (6.9%) respondents were given construction materials and the remaining 16 (27.6%) received different types of materials, including educational (e.g. books, pens, pencil exercise books) and clothing. Another component of socioeconomic support assessed was related to the involvement of PLWHA on different income generating activities. It was found that 50 (22.1%) respondents were supported to be involved in such activities. From the total PLWHA who were involved in income generating activities, 44 (65%) of them were involved in small business enterprises, followed by 4 (8%) respondents who obtained loans (Figure 1). Out of the total number of respondents, 144 (63.7%) of them had obtained food support at least once in the past 12 months. The type of food support given to 62 (41.9%) of PLWHAs was powder and packed oil, followed by powder only to 40 (27%) participants, packed oil only for 24 (16.2%) participants and grains and cereals to 4 (2.7%) participants. The remaining 18 (12.2%) had obtained other types of food substances as a support. It was also shown that 220 (97.3%) of the responding PLWHAs had obtained various trainings, at least one relevant to their health condition and socio-economic status within the past 5 years (Table 2).

**Table 2:** Summary of Socio-economic supports given to PLWHA in Arba Minch town and Surrounding areas, SNNPR, 2009

**Type of support**		**Frequency**	**Percent**
Material support	Yes	58	25.7
	No	168	74.3
Income generating activities	Yes	50	22.1
	No	176	77.9
Food support	Yes	148	65.5
	No	78	34.5
Different skill trainings	Yes	220	97.3
	No	6	2.7

As to the major challenges of care and support that the providing organizations are facing, financial and technical limitations were among the major constraints. A young female representative of a PLWHA association in the town explained her doubts about the limiting factors in an honest and confident way: -

"“*We would be very happy if we could have fulfilled at least the basic needs of the members of our association, but what limits us is mainly the lack of financial support and external technical assistance in planning, implementing, monitoring and evaluating different projects”.*"

Regarding resources allotted by the associations for care and support activities, almost all the PLWHA associations and NGOs working in the area report that much of their limited financial, material and human resources are allocated to the program, while governmental institutions were limited to only few components of care and support. This is reflected in the response from the head of a government health center who said:-

"*“We have one community counselor and few Voluntary Counseling and Testing (VCT) counselors who give psychosocial and medical care, in addition few of our staffs also provide ART, Prevention for Mother to Child Transmission (PMTCT) of HIV and counseling services to PLWHA, which is strictly based on external financial and material assistance, otherwise we have no money from our annual budget allocated for care and support activities.”*"

### Medical and nursing care

Out of the 226 (100%) respondents, 186 (82.3%) received services’ got access to services with the assistance of an NGO. Family planning was also given to 88 (38.9%) of them in the month before the study. As to the different forms of family planning methods given to the PLWHAs, condoms were provided to 82 (93.2%) respondents out of the 88 who received a contraceptive method. From the 226 (100%) respondents, 132 (58.4%) had access to treatment services through medical and nursing care. The various types of services provided included: Tuberculosis (TB) diagnosis examination and anti-TB drugs for 56 (42.4%) participants who had TB symptoms and treatment for other opportunistic infections for 48 (36.4%). The remaining 28 (21.2%) obtained treatment for other disases. Care provided was ethically appropriate for 19 (44%) respondents. In addition, other medical and nursing care services that the study participants obtained included bed nutritional support for 40 (95%) and 160 (70.8%) participants were on ART medication.

### Human rights and legal support

Among the various activities that need to be undertaken to support human rights of PLWHA, we identified assistance to cope with stigma and discrimination, co-planning to help them improve their quality of life and attempting to secure equal opportunities as activities that were being provided in the area. Among the 226 (100%) participants, other than the attempts made to alleviate stigma and discrimination to 144 (63.7%) participants, almost all of the above three activities were provided to less than half of the PLWHA. Only 72 (31.3%) of them were involved in developmental activities and other type of activities, while 76 (33.3%) of them were involved in planning for improvement of quality of life, and the remaining 106 (46.9%) were helped to get equal access to services at the work place or at social facilities (Table 3). Given its fundamental importance, it was interesting to find that none of the associations that were included in this study was providing human rights protection and guidance or legal support to their clients.

**Table 3:** Summary of Psychosocial, legal and human rights support given to PLWHA in Arba Minch town and Surrounding areas, SNNPR, 2009

**Type of support**		**Number**	**Percent**
Human rights and Legal Support	Alleviation of stigma and discrimination	144	63.7
	Involvement of PLWHA on different developmental activities	72	31.3
	Planning to improve quality of life	76	33.3
	Helped to get equal access to opportunities (employment or health services)	106	46.9
Psychosocial support	Follow up PLWHA cases	10	4.4
	Counseling services	128	56.6
	Spiritual support	118	52.2
	Community moral support	104	46.0

A representative from local NGO explained this: -

"*“Even if we are trying to alleviate the problems of PLWHA through the provision of care and support services, we are not that much working in terms of the provision of human rights protection and legal support, we need to work much in these aspects.”*"

### Psychosocial support

Participants reported almost no psychosocial support. Only 10 (4.4%) participants had their cases followed-up and 128 (56.6%) participants said they had obtained counseling services. More than half of our sample, 118 (52.2%), reported spiritual support from religious groups, while the remaining 104 (46%) were given community moral support as part of psychosocial support. The major drawback of care and support activities is the lack of evident referral and linkage system between different organizations. Four of the organizations providing care services didn’t have any referral forms for inter-organizational communication, except for the existence of referral tools which were non-specific and were mainly used for intra-organizational communication.

All the organizations agreed that it would be better if additional financial and external technical assistances were provided. They also underscored the importance of improving information dissemination among associations through the organization of assemblies or committees that aimed to discuss the challenges and improvement of service quality.

## Discussion

Sepulveda and colleagues estimated that each year, at least one in 200 people in the five African countries that they included in their study (Botswana, Ethiopia, Tanzania, Uganda, and Zimbabwe) needed palliative care at the terminal stages of HIV/AIDS or cancer [21]. This study assessed care and support activities provided to PLWHAs by various institutions in the town of Arba Minch, Ethiopia. The current study showed that 141(62.4%) participants who received care and support services were females and between 26–30 years of age. Our findings underscore that almost all of the care and support activities available are not adequate and not well organized. This is evidenced by the proportion of PLWHA who received care and support as well as by the various components of the care and support activities.

PLWHA have diverse and complex needs in terms of access and provision of care and support services. From our sample of 226 (100%) participants, only a quarter obtained some type of material support. This reflects the limited access to care and support services that is prevalent, and points out to the urgent need to scale-up these services in this region. Scaling-up services could potentially have a significant impact in the social skills of PLWHA and could further impact productivity within their communities. Furthermore, technical support and material support should be increased to adequately provide services and to identify barriers and challenges for service provision and to develop plans to address them.

According to WHO Palliative Care Project in Africa, the basic palliative care package should include analgesics and drugs for symptomatic relief, food and family support [21]. In our study, we found that approximately half of the participants were receiving some type of food support, which was limited to basic cooking materials. This underscores the need to scale-up programs that provide these basic services and to develop mechanisms to ensure that food reaches target populations, while evaluating the impact of such services.

Care should include psychological, social, and economic support as well as broad based medical care incorporating nutritional guidance, prevention and treatment of opportunistic infections and palliative care [22]. In our study setting, local health facilities emphasized medical and nursing care; however, this was not according to the continuum of care and was entirely dependent on external financial support. Given the limited number of associations providing this services in the area, current and new methods of communication should be strengthened and implemented to prevent overlap and make service provision of better quality and more efficient. Services should also include home-based care and support, which was reported as preferable by PLWHA who participated in this study. Previous research conducted in Jimma, Ethiopia showed that home is an ideal place for medical, social and psychosocial care and support [15]. These findings are supported by research in East Africa that explored quality of care and unmet needs of people requiring palliative care [21,23,24].

Important issues that need to be addressed are the need for food, the severe financial constraints on the family and caregivers, the need for training of family caregivers, lack of psychosocial support, and social isolation due to the stigma attached to a diagnosis of HIV/AIDS [25]. A study carried out in Ghana showed that even though PLWHA have better health outcomes with ART, they still face psychological isolation and condemnation from their family, friends and society because people around them are aware of their HIV status [26]. Stigma and discrimination can disrupt efforts to receive care and support services. These experiences can have a long-term impact on recovery, and also on the health and psychological status of PLWHA [27]. A study carried out on utilization of ART in Ethiopia reported that female accessibility to this service remained low due to the stigma attached to seeking treatment [28], emphasizing the need for stronger community and home based care programs [29]. Various community-based programs, which can complement the institution-based approach pursued by the government, focus on community and home-based care and support [30-32].These approaches are currently being implemented by GOs and NGOs [30]. By 2006, home and community-based care programs that are improving benefits and quality of life of PLWHA were being implemented in 14 major towns in Ethiopia [30]. Our study also showed that medical and nursing care, including family planning, preventive therapy, Sexually Transmitted Infections (STI) diagnosis and treatment, bed-based nutritional support, laboratory support, and palliative care are given less attention by care givers. It was said that families and the community should first accept the patient and respect them, and not judge or ostracize them [33]. Protection of human rights and the provision of legal support, as well as psychosocial support are neglected by health facilities or by organizations that have, as their fundamental purpose, to provide these services. Similar findings have been documented in needs assessment carried out in Addis Ababa, Ethiopia, which showed protection of legal and human rights of PLWHA and AIDS orphans as one of their most important concerns [34]. Policymakers can also influence the quality of health care through legislation, regulation and accreditation of minimum standards. Legislation can protect the rights of people with chronic conditions. The promotion of human rights occurs, in part, through access to health care and voluntary treatment. Regulatory frameworks can be developed and enforced that protect healthcare institutions and workers. Anti-discrimination laws for housing and employing persons with chronic conditions can also be adopted [35]. Stigma was a recurrent issue that arose in the interviews that were conducted. It was evident that youths had been denied many rights related to health. We concluded that young people living with HIV/AIDS need comprehensive care based on a human rights approach [36]. The other problem that was given little attention but can have a major impact in the implementation of care and support activities is the lack of client referral and linkage between caregivers and organization. This is mainly reflected by the inadequate range of services and their redundancy. We recommend that this failure can be overcome by improving coordination between organizations through the creation of a local assembly that would gather heads of local organizations, care-givers, community and PLWHA.

Some of the strengths that were identified in our study were that it was conducted to assess care and support services both at the community level and to PLWHA association, this design can provide us with more robust information than relying on either of them individually. Furthermore, we employed qualified and trained data collectors who had adequate and relevant educational background and work experience. We also utilized home based care givers for the household survey to collect data from PLWHAs who are bed ridden and are getting home based care and support. Some of the limitations that we identified in our study were that the study relied on information dating back up to one year of care and support which, may not exactly reflect the recent care and support services provided, recall bias might affect the information obtained from the participants and critically sick respondents, the potential for a selection bias in HIV palliative care evaluation was demonstrated in a study that found patients reported less anxiety, and fewer spiritual problems than in providers’ assessments [36]. Patient self-reports are also subject to bias because of more unwell patients being unable or unwilling to participate.

## Conclusions

Although care and support services are new in the area, it can be said that care and support activities provided are minimal and most of the respondents are not getting the palliative care services that they need. Psychosocial, legal and human right services are widely neglected or are not given due attention by almost all caregivers as part of the standard of care and support. Modest achievements were observed in the provision of medical and nursing care services including: palliative care, bed-based nutritional support, family planning, preventive therapy and others. Organizations, external donors and concerned governmental bodies providing care and support services have to maximize their financial, human and material supports to a level that best fits the needs of PLWHAs. It is up to all concerned organizations to utilize the current prospects and opportunities available to maximize their activities and bring tangible changes on the life of PLWHAs.

## Competing interests

All authors declare that they have no conflict of interest associated with the publication of this manuscript.

## Authors’ contributions

TAZ, YY conceived and designed the study and collected data in the field, performed analysis, interpretation of data, and draft the manuscript. SD assisted with the design, interpretation of data and the critical review of the manuscript. SB participated in design and helped to draft the manuscript and critically reviewed the manuscript. KD helped to draft the manuscript and critically reviewed the manuscript. HRE participated in critically reviewed the manuscript. All authors approved and read the final manuscript. All authors participated in critical appraisal and revision of the manuscript.

## Disclaimer

The views, the findings and conclusions represented in this article are those of the authors and do not necessarily represent the official views of their institutions where they affiliated.

## Pre-publication history

The pre-publication history for this paper can be accessed here:

http://www.biomedcentral.com/1472-684X/11/8/prepub
